# Twitter Sentiment Geographical Index Dataset

**DOI:** 10.1038/s41597-023-02572-7

**Published:** 2023-10-09

**Authors:** Yuchen Chai, Devika Kakkar, Juan Palacios, Siqi Zheng

**Affiliations:** 1 Sustainable Urbanization Lab, Massachusetts Institute of Technology, Cambridge, MA 02139 USA; 2 Center for Geographic Analysis, Harvard University, Cambridge, MA 02138 USA

**Keywords:** Interdisciplinary studies, Society

## Abstract

Promoting well-being is one of the key targets of the Sustainable Development Goals at the United Nations. Many national and city governments worldwide are incorporating Subjective Well-Being (SWB) indicators into their agenda, to complement traditional objective development and economic metrics. In this study, we introduce the Twitter Sentiment Geographical Index (TSGI), a location-specific expressed sentiment database with SWB implications, derived through deep-learning-based natural language processing techniques applied to 4.3 billion geotagged tweets worldwide since 2019. Our open-source TSGI database represents the most extensive Twitter sentiment resource to date, encompassing multilingual sentiment measurements across 164 countries at the admin-2 (county/city) level and daily frequency. Based on the TSGI database, we have created a web platform allowing researchers to access the sentiment indices of selected regions in the given time period.

## Background & Summary

Subjective Well-Being (SWB) is commonly defined as the combination of reflective cognitive judgments and emotional feelings in ongoing life^1^. SWB indicators have been increasingly used by researchers and policymakers as measures of life satisfaction to complement traditional objective development and economic metrics^2^. In the meantime, research centred around SWB has grown enormously in recent years^1^. Studies in this field have documented strong correlations between SWB and important human outcomes^3^, such as health and longevity^4^, social relationships^1^, and earning^5^.

Given the importance of SWB, researchers have been putting a lot of effort into measuring SWB, mainly employing self-report interviews and surveys^6^. For example, Gallup surveys collect well-being indicators worldwide^3^. Similarly, household panels such as the PSID in the US^7^, SOEP in Germany^8^, and the BHPS in the UK^9^ incorporate SWB measures in their questionnaires. More recently, there have been efforts to map the impact of the COVID-19 pandemic on SWB. For instance, Patrick *et al*. (2020) conducted a national survey in the US to measure satisfaction with many aspects of daily life during the COVID-19 period^10^. Although survey methods are effective in quantifying SWB, they have scalability problems, as well as significant time delay and high implementation cost^11^. Such limitations are especially pronounced when researchers or policymakers try to make timely evaluations of well-being changes in response to unexpected events (e.g., epidemics or climate disasters). There is a growing interest in developing more efficient methods that allow researchers to monitor instant well-being and trace back to history with high spatial-temporal granularity.

The rising adoption of social media platforms worldwide, together with the advances in Natural Language Processing (NLP) techniques, provides a new and valuable complement. Every day, active social media users create content through these platforms, generating useful traces of their attitudes, beliefs, and feelings at every moment^12^. For example, Twitter, one of the major social media platforms, has over 330 million monthly active users across the world^13^. While this scale of data was traditionally impossible to analyse, recent developments in NLP have made it possible to automatically extract sentiment information from unstructured social media posts using high-performance computing infrastructures.

Researchers in the field of NLP have recently developed sentiment analysis algorithms to quantify the affective states from texts^14^ and validated it to have strong correlations with SWB^2^. The existing studies have used different methods to extract overall scores of positive and negative emotions through either word-level or data-driven methods^2^. For example, Passi and Motisariya (2022) measure the public sentiment toward political leaders using Linguistic Inquiry and Word Count (LIWC)^15^. Schwartz *et al*. (2019) leverage the Hedonometer Index to investigate the evolution of expressed happiness before, during, and after visits to San Francisco’s urban park system^16^. Lyu *et al*.^17^ uncover the sentiment trend of the Chinese population during the peak period of COVID-19 using Bidirectional Encoder Representations from Transformers (BERT)^17^. Although there has been significant progress in sentiment analysis, the existing studies either focus on a specific topic/event or are limited to narrow temporal and spatial extents.

**Fig. 1:**
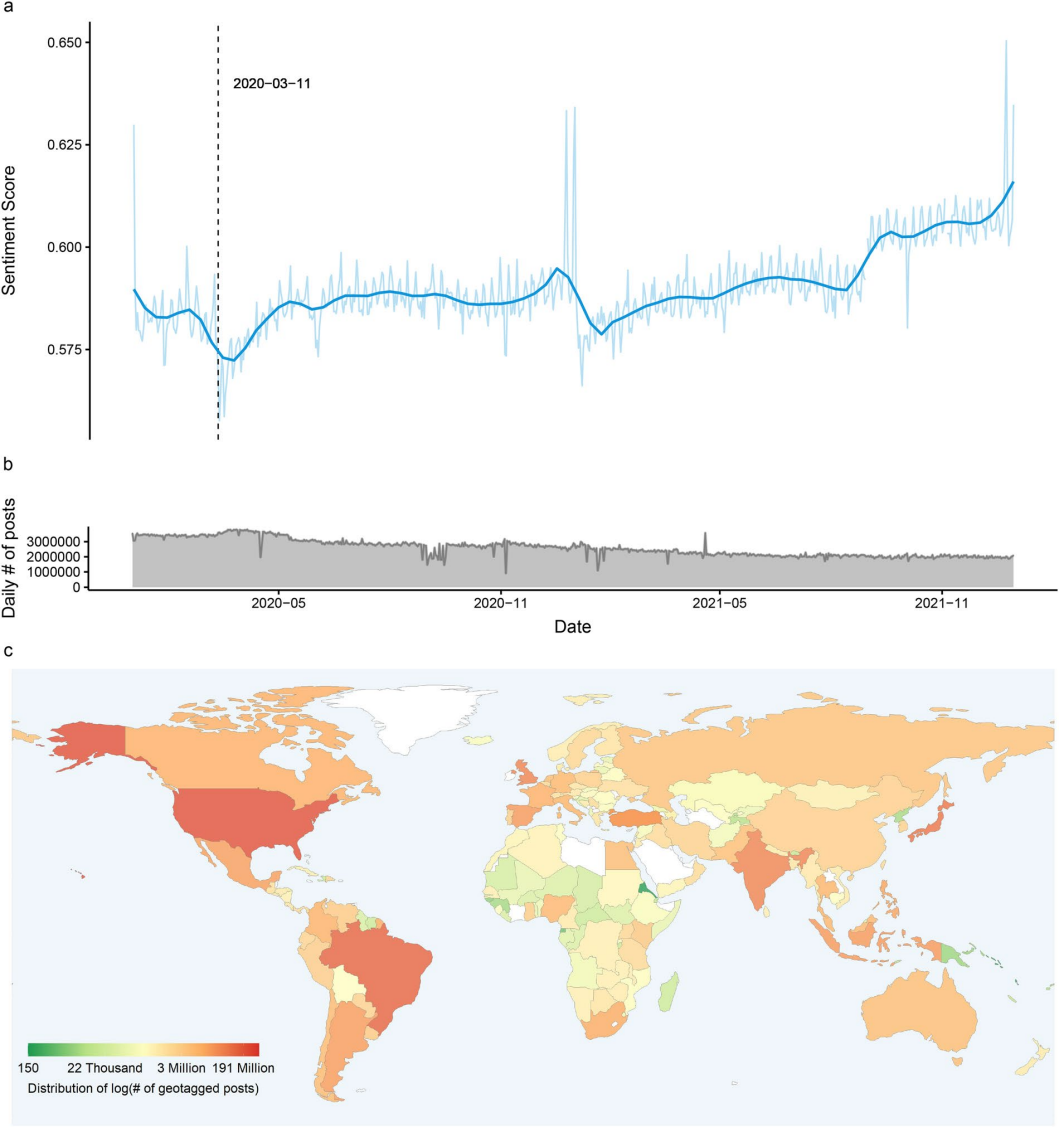
(**a**) Line graph shows the daily sentiment index for the whole world from January 1st, 2020, to December 31st, 2021. In general, the aggregated sentiment is floating around 0.6 and is gradually increasing after being impacted by COVID-19 on March 11th, 2020 (WHO declared the COVID-19 outbreak a global pandemic). (**b**) The area plot displays the daily number of geotagged posts used for generating the sentiment index during 2020 and 2021. On average, there are 2.64 million geotagged posts collected daily during these two years. (**c**) The world map illustrates the spatial distribution of collected geotagged posts at the country level for 2021. Countries with color closing to red generate more tweet traffic while color closing to green generates fewer tweets. Among all countries, the USA has the most geotagged tweets taking up 24% of the total traffic, followed by Brazil (14%) and Japan (9%). The uneven tweet generation is due to many reasons such as the number of users/population size or application preference (e.g., users in China prefer using Sina-Weibo).

This project aims to develop a comprehensive Twitter sentiment geographical index (TSGI)^18^, an expressed sentiment database having SWB implications, that can provide a high-resolution and large-scale complement to traditional survey measures of SWB. The Sustainable Urbanization Lab of MIT trains a BERT-based multi-lingual sentiment classification model based on a labelled database and applies it to a global geotagged Twitter archive^19^ (containing 10 billion posts) from the Center of Geographic Analysis (CGA) of Harvard. We impute a sentiment score, defined as the probability of a post being classified as a post with a positive mood, for every post and aggregate them to multiple administrative levels (i.e., city/county, state, and country) daily. Figure 1 below shows the global sentiment trend, daily count of tweets, and spatial distribution of tweets on the country level for a sample period. To validate our data, we conduct several analyses: (1) We test the model accuracy on an additional multi-lingual sentiment dataset; (2) We conduct an analysis to investigate the geolocation origin of the posts with different languages; (3) We replicate sentiment classification methods including dictionary-based and bag-of-word based and compare the performance of these models on the test dataset; (4) We implement a language usage test on 20 million randomly selected tweets with word shift visualization. In addition, to facilitate public access to our generated indices, we develop a freely accessible web platform available to the entire research community, so that researchers can easily view the evolution of global and regional sentiment.

This dataset has been partly used by a previous work which is published in Nature Human Behavior^20^. The work documents the causal relationship between the outbreak of COVID-19 and a steep decline in expressed sentiment followed by a slower recovery in multiple countries. This study provides an example of how our dataset can be utilized to investigate global major events.

Our primary contribution lies in the development of the most comprehensive open-source geotagged Twitter sentiment database to date. In contrast to previous studies that used the Twitter sentiment for a specific research question within a particular country, our database offers open access and several advantages: (1) Model performance: By employing BERT as the embedding method, we take sentence context into account to improve the accuracy of downstream classification tasks (see the quantitative performance comparison between our approach and other traditional approaches using our Twitter data); (2) Multilingual computation: We utilize a consistent method for multilingual sentiment computation on a global scale, enabling cross-country comparisons across over 164 countries; (3) Temporal coverage: Our dataset encompasses the most recent five-year period, allowing researchers to examine the sentiment impacts of the most up-to-date social challenges and policies. We expect that our TSGI database will be able to support in-time investigations of expressed sentiment alterations for researchers across disciplines and be a valuable complement to traditional surveys to provide SWB insights.

## Methods

### Twitter data collection

Geotweet Archive v2.0^19^ (Archive): To generate our global sentiment index, we retrieve raw tweet data from Archive, a project at the Center for Geographic Analysis (CGA) at Harvard University. CGA maintains the Geotweet Archive v2.0, a global record of tweets spanning time, geography, and language. The primary purpose of the Archive is to make a comprehensive collection of geo-located tweets available to the academic research community. The Archive extends from 2010 to 2023. More information on this dataset is available (https://dataverse.harvard.edu/dataset.xhtml?persistentId=doi:10.7910/DVN/3NCMB6). Tweet IDs can be requested via the request form (https://gis.harvard.edu/geotweet-request-form).

The tweets in the archive are harvested using the Twitter Streaming API which allows users to stream Tweets in real-time. Only tweets that carry one or both of the spatial attributes (Coordinate and Place) are included in the Archive. Approximately 1–2% of all tweets contain such geographic coordinates although this percentage needs verification and may vary over time^21^.

Twitter has two attributes/objects Coordinates and Place which are associated with the spatial location of the tweet. Each of these is described in detail below:

- Coordinates: This attribute represents the geographic location of the Tweet as reported by the user or client application. The inner coordinates array is formatted as geoJson with longitude first, then latitude.
- Place- Twitter place attribute when present indicates that the tweet is associated with a place. It is the geographic place as defined by the user and is usually a town name. A bounding box is determined by Twitter based on this field. We assume the centroid of this Bounding Box as the coordinates when actual GPS coordinates are not present in the tweet. Further, we find the radius of the circle of this BB to estimate the spatial error associated with the coordinates.

The key fields for location signatures are described below:

- Latitude and Longitude- Every tweet has a latitude and longitude field which is derived from either:
  - Twitter Coordinates objects or
  - Calculated using the centroid of Bounding Box based on Twitter’s place object

- GPS: Flag for whether the coordinates of tweets are taken from GPS or Place name-based bounding box. If the tweet coordinates are from GPS, then this field is marked “Yes” otherwise “No”. When both are present, the GPS coordinate takes priority. This helps us determine if the tweet coordinates are the actual GPS coordinates from the tweet or derived coordinates based on the place name.
- Spatial Error- Every tweet is marked with a spatial error field which is very crucial in interpreting the location of the tweets. This is an estimate of meters horizontal error for the tweet coordinates. We have assumed a 10 m spatial error for GPS coordinates whereas the error for place name-based coordinates is calculated as the Radius of a circle with the area of the bounding box.

The count of tweets mentioned in the manuscript refers to tweets harvested directly from Twitter with one or both of the spatial attributes mentioned above. We do some pre-processing of tweets to add a few additional fields/attributes (particularly spatial attributes) to support our research. However, this pre-processing only added new fields to each tweet and did not change the total number of tweets harvested. To ensure the data quality, we reserve the data starting from 2019, which contains an overall 4.37 billion tweets until the end of December 2022. On average, 2.99 million geotagged tweets are collected every day.

### Twitter data cleaning

Geotweets from bots’ sender names are not generated by a human with a mobile device since they are randomly scattered across the globe. After a thorough analysis of our data in January 2018, we discovered several automated tweet-bots which generate tweets with randomly spoofed coordinates. These bots appear to be randomly distributed spatially. We provide a list of the most commonly occurring bots in Table 1 below. These bots make up less than 1 percent of harvested Geotweets.

**Table 1:** List of major automated tweet bots discovered as of January 2018.

Index	Bot name	Index	Bot name
1	googuns_lulz	6	AL_FiN_07839216 _grammar_ equivocagent
2	googuns_staging	7	mozatsubot
3	googuns_prod	8	recentideas
4	MarsBots	9	seq82
5	autoRNG	10	kaikkisanat

Before feeding the Twitter text data into the language model, to ensure the quality of the results, we take several pre-processing steps. Following Pradha *et al*.^22^, (1) we remove any URL from the tweet since they provide no information to determine the sentiment polarity; (2) given that the chosen labelled training dataset (Sentiment 140) has stripped out all emoticons to prevent potential overfitting^23^, we also remove emojis from the tweets to ensure the same format of our data; (3) we replace varying user mentions with a fixed format of string (@user) to notify the model about mentioning; (4) we remove any non-alphanumeric words from the original tweets.

**Fig. 2:**
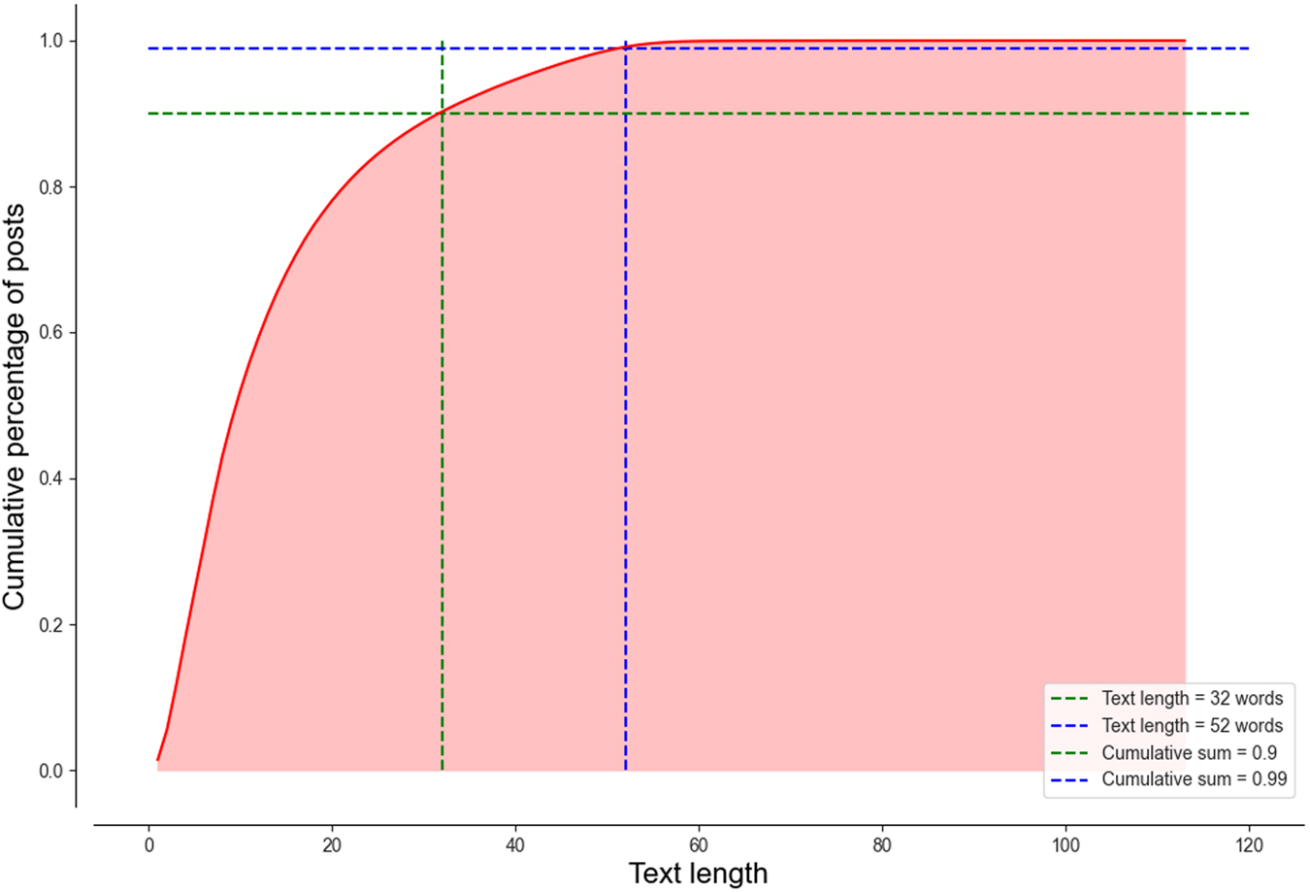
The text length distribution of a randomly selected 20 million geotagged tweets generated in 2021 from the Archive. The figure shows that 90% of geotagged posts have less than 32 words while 99% of geotagged posts have less than 52 words.

In addition to the steps mentioned above, another step we take is to truncate the sentence. A sentence with more words contains more information. However, it requires a larger memory to store the information and a longer time for the language model to process it. According to our statistics on a randomly selected 20 million geotagged tweets generated in 2021 from the Archive, 90% of posts have a length of fewer than 32 words, and 99% of posts have a length of fewer than 52 words (Fig. 2). To balance the performance and efficiency, we choose to discard any inputs beyond 52 words.

We apply these steps to all text involved in this project to assure consistency between model training, testing, and predicting.

### Sentiment analysis

**Fig. 3:**
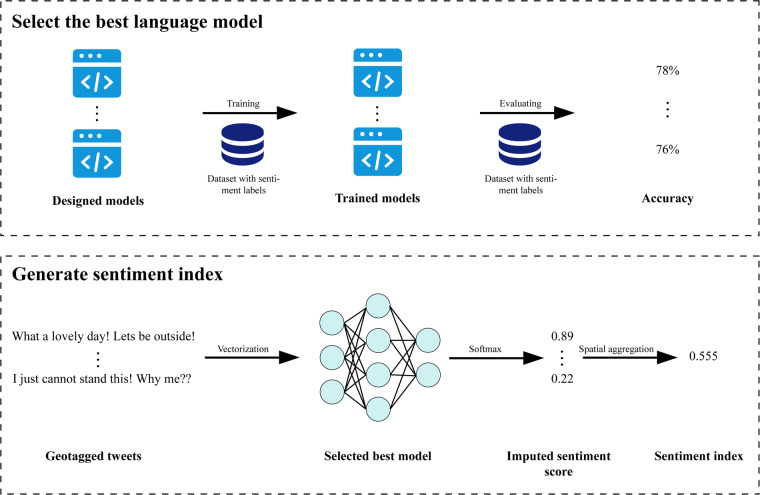
Schematic to understand the logic of sentiment index generation. Upper part shows the steps to train sentiment models and select the one with the best performance. After designing the structure of a neural classifier, we train the model using 80% of the Sentiment 140 dataset and evaluate the performance of the remaining 20%. All models are trained on the same training dataset and evaluated on the same test dataset to ensure consistency. Lower part describes the steps to generate a local sentiment index. We input the vectorized geotagged tweets into the selected best model. Then, we assign a value between 0 and 1 to represent the sentiment score for each tweet using the SoftMax function. Finally, we aggregate the tweet level sentiment score to county/city, state, or country level to represent the local sentiment index.

The imputation of sentiment scores for each Tweet is made in two separate steps^24^. First, we create semantic representations by extracting contextual information from text data. Next, we train and select the classifier which performs the best on the testing dataset to decide the sentiment polarity for the training data (i.e., positive sentiment and negative sentiment). Since assigning a dummy sentiment label to each tweet ignores the delicate difference between tweets, we leverage the SoftMax^25^ function to assign a score between zero and one to represent the tweet sentiment intensity (see Fig. 3). We describe each step in the following sections.

#### Creating the semantic representations for text data

Converting human-readable text into a machine-readable numerical sequence is known as text representation^26^. Traditional dictionary-based methods usually encode sentences into a list of 1’s and 0’s based on whether a list of given words is in the sentence or not^27^. However, the representations generated by these methods neglect synonyms, word order, and sentence construction, leading to high sparsity problems and resulting in poor performance for the downstream tasks^28^. Those methods are also constrained by the availability of dictionaries in certain languages. In 2018, Google released the BERT model which reads the text as sequences of words and uses a transformers architecture to assign contextual embeddings to words based on their nearest neighbors^29^. This machine-learning technique ensures that words with similar semantic meanings will have similar fixed-length representations, overcoming the previously mentioned weakness. This feature also allows researchers to finetune the model using a rich-resource language dataset and then apply it to other lack-of-training-resource languages. Many researchers have documented that the sentiment analysis utilizing the pre-trained BERT model outperforms traditional methods across languages^30,31^.

For this project, we leverage a modified BERT model, i.e., Sentence-BERT (S-BERT)^32^, with a pooling layer that combines all the word embeddings into a single representation indicative of the entire sequence of words. We import the model weights from “stsb-xlm-r-multilingual”, a model pre-trained on Stanford Natural Language Inference (SNLI) corpus^33^, Multi-Genre Natural Language Inference (MultiNLI)^34^ corpus, and Semantic Textual Similarity (STS) benchmark dataset^35^, allowing to encode text for more than 50 languages. The selected model takes the truncated pre-processed input and generates a vector of fixed size 768 (pre-defined by the model) representing the entire tweet.

#### Training sentiment classifier

**Fig. 4:**
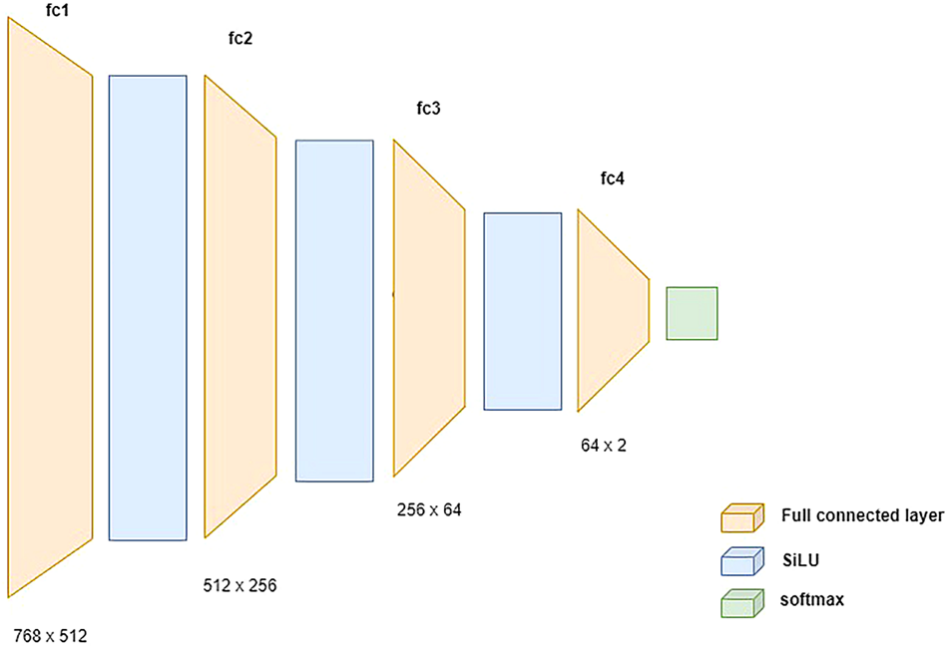
Designed dense network architectures for sentiment classification.

Sentiment classification is the second step in the implementation of sentiment analysis. It receives the input of text representations and assigns a sentiment label (positive or negative) to each of them. A wide range of models including logistic regressions and neural networks can be fitted on the labelled training dataset and give a prediction on an unlabelled Twitter post^36^. For this sentiment classification task, we specifically design a four-layer dense neural network with the Sigmoid Linear Unit (SiLU)^37^ as the activation function (Fig. 4).

The training and testing data we use is Sentiment 140, an English-language sentiment-labelled dataset containing 1.6 million Twitter posts^23^. The dataset was constructed by imputing sentiment on every tweet based on the occurrence of positive or negative emojis with the text. We first apply the pre-processing steps described in the previous section, then compute the S-BERT embeddings for every observation in the Sentiment 140 dataset, train a classifier on 80% of the data (training set), and test the classifier on the remaining 20% of the data (testing set). In the end, our best model is trained using an SGD optimizer with 60 epochs achieving 83% accuracy on the testing set using the aforementioned neural network structure. As the baseline, a logistic regression model without any penalty achieves 81% accuracy on the testing set.

#### Assign sentiment intensity

Labelling a single tweet or a group of tweets with a continuous score between 0 and 1 is defined as sentiment intensity assignment, where 0 represents the most negative sentiment and 1 represents the most positive sentiment. Simply generating a dummy sentiment classification of each tweet is not granular enough to capture the intensity of sentiment. Therefore, we decide to assign a sentiment score at the tweet level to achieve a better proxy.

The last dense layer of the neural network outputs two unbounded values representing the weight of positive and negative categories. Then, following He *et al*., (2017)^38^ who applies SoftMax to a computer vision classification task, we add a SoftMax layer to convert the outputs of the last dense layer into a value between 0 and 1, representing the probability of the tweet to be categorized into positive sentiment class. We use this likelihood as the sentiment score on the tweet level.

### Aggregating tweet-level sentiment scores

In this subsection, we describe how we aggregate the sentiment of Tweets to construct sentiment indices. We define the boundary of the administrative area for each tweet and conduct spatial intersection to find the corresponding spatial belongings of each tweet. Finally, we aggregate the sentiment scores to sentiment indices based on their spatial belongings.

We retrieve the global administrative boundary data from the Database of Global Administrative Boundaries (GADM), which contains a minimum mapping unit of ADMIN 2 (county/city) with the global scale and is updated frequently. For our TSGI, we use GADM version 3.6 for the spatial analysis which was the latest build when we downloaded the data. In total, GADM contains 256 distinct ADMIN 0 areas (country/region); 3638 distinct ADMIN 1 areas (state/province), and 46782 ADMIN 2 areas (county/city).

Using the acquired GADM vectorized data, we conduct spatial intersection for each tweet using Heavy.ai^39^ on Harvard FAS Research Computing infrastructure. For each tweet, we extract the spatial information including ADMIN 0, ADMIN 1, and ADMIN 2 from GADM, allowing us to build sentiment indices at different levels.

Besides the aforementioned spatial information, each tweet is labelled with the date when the tweet was generated. We then average the value of sentiment scores over location and date to build the temporal local index. To enable researchers to aggregate the sentiment scores on different scales (different spatial levels such as metropolitan areas; different temporal levels such as weekly or monthly periods), we attach a field indicating the number of tweets for every sentiment index record in our dataset.

## Data Records

The aggregated multi-level (Globe, country, state, and county/city) daily sentiment indices are available at 10.7910/DVN/3IL00Q^18^. Multiple comma-separated values (CSV) files are under the folder with the information shown in Table 2, each for sentiment indices in each year starting from 2019. Users can access data for free and use any level of the index to best fit their purpose of usage.

**Table 2:** The variable and their explanations for the county-level daily sentiment indices.

Entry	Variable	Notes on variable
1	DATE	Date for the sentiment index
3	NAME_0	Country name
5	NAME_1	State/Province name
7	NAME_2	County/City name
8	SCORE	Mean sentiment of all posts within the geospatial range on a given date
9	N	Number of posts within the geospatial range on a given date

Moreover, a visualization of the historic sentiment indices is available on https://www.globalsentiment.mit.edu/dataset, based on the latest version of the sentiment analysis model.

## Technical Validation

### Model validation on an external dataset

Since multilingual Sentence-BERT can convert sentences in different languages with similar meanings into similar vectors, it allows us to train the model on a rich-resource language and then apply it to other languages. In our case, the model is trained on the first 80% of Sentiment 140, a pure English-language dataset that achieves 83% accuracy on the rest of the 20%. We wish to evaluate the performance of our model in different languages.

We retrieve a multilingual sentiment dataset for 15 languages^40^. There are 1.6 million tweets id included in this dataset together with their sentiment label in negative, neutral, and positive classes. We rehydrate tweets text using Twitter API^41^; clean the text using the same pre-processing techniques; vectorize the text using the same Sentence-BERT model and apply the best-trained model on positive and negative tweets to get sentiment labels to every recovered tweet. Table 3 shows the performance of the model across 15 languages. Overall, our model achieves 71.4% accuracy on this corpus.

**Table 3:** Model accuracy on an external dataset.

Dataset	Size	Share in 2021 tweet	Accuracy	Precision
Albanian	22,126	—	69.2	83.6
Bosnian	13,621	—	74.7	74.6
Bulgarian	12,320	0.02%	72.1	73.1
Croatian	45,505	—	79.6	82.3
English	22,844	38.66%	78.6	77.0
German	29,705	0.77%	74.5	73.9
Hungarian	26,880	0.06%	75.5	89.5
Polish	84,758	0.41%	71.7	73.7
Portuguese	34,539	13.45%	57.9	48.6
Russian	23,751	0.82%	71.3	67.0
Serbian	21,311	0.04%	63.4	53.2
Slovakian	37,021	—	77.2	80.7
Slovenian	42,978	0.05%	72.4	66.7
Spanish	81,143	12.42%	67.9	86.1
Swedish	20,068	0.20%	67.7	58.4

Since the language of the tweet is not evenly distributed^42^, to estimate the general accuracy of our model on the actual data, we conduct additional analysis. Of all the data we have in 2021, 38.66% of geotagged tweets are in English, followed by Portuguese (13.45%) and Spanish (12.42%). In total, these 15 languages cover 66.90% of all tweets in 2021. Taking the share of language as the weight, the adjusted model accuracy for the languages is 72.2%.

### Language usage validation on a raw dataset

**Fig. 5:**
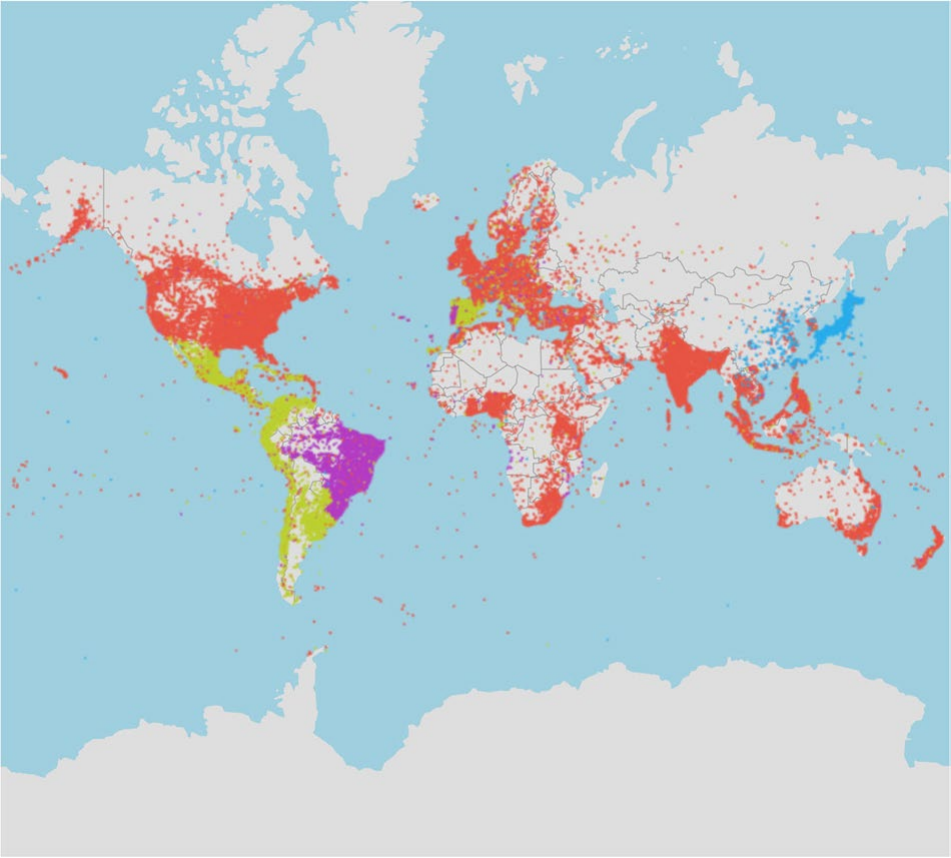
Spatial distribution of top 4 most used languages for 50 million tweets between January and February 2023 (English: red, Portuguese: purple, Spanish: green, Japanese: blue).

To validate language use and their location we analysed the latest 50 million tweets between January and February 2023. We checked the language distribution of tweets within our sample. As shown in Table 4, the top four languages already account for 70% of the geotagged tweets. We plot the spatial distribution of tweets for these four languages. We further plotted the spatial distribution of tweets in these top languages. It can be seen from Fig. 5 that English tweets are distributed across the globe with the highest concentration in the US, Europe, and South East Asia. The Spanish tweets are coming mainly from Mexico, Spain, Colombia, Peru, Chile, and Argentina. The Portuguese tweets are coming mostly from Brazil. The Japanese tweets are concentrated in Japan. Thus, it is validated that the languages of tweets correspond well with the geographical areas where they are the predominant language.

**Table 4:** Language distribution of tweets.

Language	# of tweets in 50 million sample	Percentage of tweets in 50 million sample
English (en)	16,618,957	35.81%
Spanish (es)	5,897,099	12.71%
Portugese (pt)	5,300,609	11.42%
Japanese (ja)	4,881,423	10.52%
Arabic (ar)	1,581,877	3.41%
Indonesian (in)	1,370,835	2.95%
Turkish (tr)	1,340,662	2.89%
Hindi (hi)	931,777	2.00%
Others	9,146,503	18.29%

Recognizing that smaller languages are more susceptible to biases in spatial representation, we conducted a spatial concentration analysis for several such languages, namely Tibetan, Armenian, and Sindhi. Although the number of tweets these languages is fewer than 5000 within our 50 million sample, we found that around 90% of these tweets are concentrated in countries where the corresponding languages are spoken (Table 5). This observation provides further assurance of the validity of our spatial mapping of geotagged tweets.

**Table 5:** Spatial distribution of tweets in Tibetan, Armenian, and Sindhi.

Language	# of tweets in 50 million sample	Country	Number of tweets	Ratio
Tibetan	648	China	631	97.38%
Armenian	1621	Armenia	1555	95.93%
Sindhi	2419	Pakistan	2059	85.12%

### Model performance against other sentiment methods

To demonstrate our model’s performance, we benchmark the performance of our sentiment classification using BERT as the word embedding approach against the most commonly used dictionary and bag of words sentiment methods employed in other studies: LIWC^43,44^, VADER^45^, and Bag of words^46^. As demonstrated in Table 6, our trained model significantly outperforms other methods on the testing dataset in terms of both accuracy rate and F1 score.

**Table 6:** Sentiment model performance on the testing dataset.

Index	Embedding Method	Classification method	Accuracy	F1 score
1	LIWC	Voting	0.288	0.394
2	VADER	Voting	0.285	0.378
3	Bag of words (5000 features)	MultinomialNB	0.769	0.770
4	Bag of words (5000 features)	LinearSVC	0.791	0.788
5	BERT	Logistic Regression	0.808	0.810
6	**BERT (our paper)**	**Neural Network**	**0.829**	**0.829**

The performance improvement depicted above mainly focuses on expressed sentiment. Our expressed sentiment database provides useful information for investigating the spatial and temporal variations in expressed sentiment across regions, with the understanding that expressed sentiment has a correlation with underlying subjective well-being variations. Jaidka *et al*. (2020) demonstrated the validity of Twitter sentiment by correlating it with the Gallup-Sharecare Well-Being Index survey, showing that adjusted LIWC or data-driven sentiment analysis models have robust correlations with regional well-being. The BERT model we employ has shown performance improvements over dictionary-based methods like LIWC and hedonometer (Devlin *et al*., 2018; Tanana *et al*., 2021). However, no research to date has specifically tested the correlations between BERT sentiment indices and well-calibrated subjective well-being surveys. As we lack access to global subjective well-being surveys, we instead demonstrate convergent validity by examining the correlation between our TSGI index and the multilingual Hedonometer Happiness trend across languages. Hedonometer Happiness is a widely accepted happiness measure based on the bag-of-words approach to reflect the general sentiment trend on Twitter by language without the geotagged information. As shown in Table 7, our generated TSGI exhibits positive and significant correlations with the Hedonometer Happiness trend.

**Table 7:** Correlation in temporal trends of Twitter sentiment between TSGI and Hedonometer.

Year	English	German	Spanish	Portuguese
2019	0.350	0.230	0.195	0.172
	(0.000)	(0.000)	(0.000)	(0.000)
2020	0.681	0.637	0.704	0.659
	(0.000)	(0.000)	(0.000)	(0.000)
2021	0.796	0.521	0.532	0.478
	(0.000)	(0.000)	(0.000)	(0.000)
2022	0.361	0.402	0.405	0.212
	(0.000)	(0.000)	(0.000)	(0.000)

Note: Pearson correlation coefficients between the TSGI index and Hedonometer Happiness Index and the corresponding P values (in brackets) are displayed for each language in each year.

### Sentiment score and word usage

According to our definition, a sentiment score represents the possibility of a tweet being classified as a post with a positive mood. Here we investigate the relationship between sentiment score and word usage to validate our method.

First, we randomly select 2.5 million posts across 2019 and 2022 respectively and filter out non-English tweets so that the readers here can better understand the content. Then, we divide the filtered dataset evenly into four quarters according to their sentiment scores. For this analysis, only the tweets with the top 25% sentiment score (top quarter) and the bottom 25% are reserved (bottom quarter). To better show the difference between the two subsets, we introduce Linguistic Inquiry and Word Count, a dictionary to aid word usage analysis^47^. It contains more than six thousand English words with human-labelled sentiment polarity tags (i.e., the word with tag 31 means that the word contains positive sentiment, and the word with tag 32 means that the word contains negative sentiment). For each word showing up in two quarters, we only count the occurrence of those words that contain positive or negative sentiment.

**Fig. 6:**
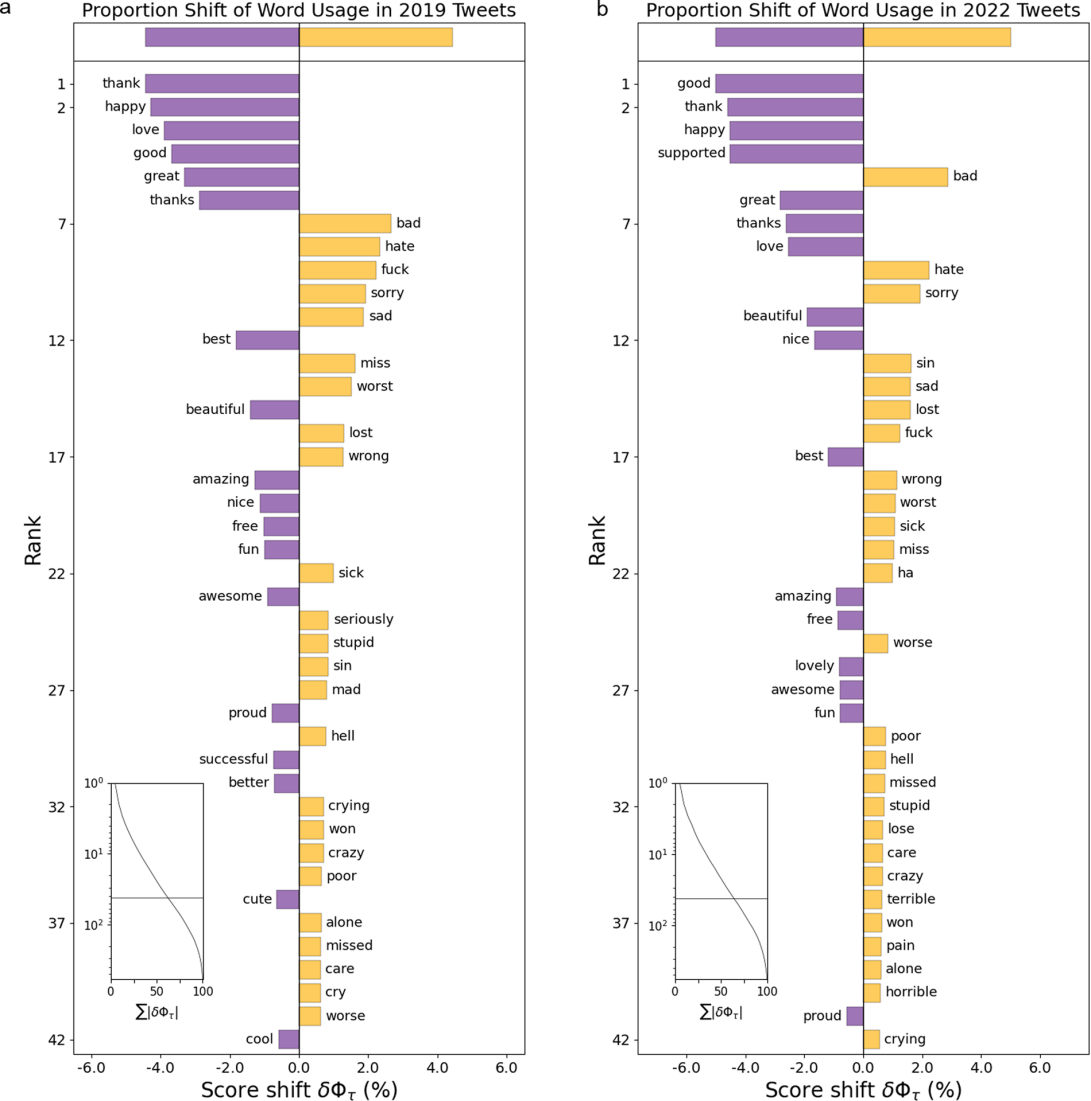
The word shift figures show the relative frequency of words in the top 25% and bottom 25% of tweets in 2.5 million randomly selected tweets in both year 2019 and 2022. Words on the left (e.g., thank, good, happy, etc.) indicate that they are relatively more common in the top quarter of tweets while words on the right (e.g., bad, hate, sorry, etc.) represent that they show up more in the bottom quarter of tweets.

Using a word shift figure^48^ (Fig. 6), we show the relative frequency change of words in the top quarter and the bottom quarter. People tend to use words on the left to convey positive moods such as “thank”, “good” or “happy”, while using words like “bad”, “hate” or “sorry” to express negative sentiments. The consistency in word usage to express sentiment across years demonstrates that our trained model can effectively capture human sentiment expression patterns, despite being trained on data from several years ago.

## Usage Notes

This dataset offers a complementary data source to investigate rich topics related to SWB. It mainly provides a detailed sentiment index spanning time and geography. To the best of our knowledge, it is the most extensive social media-expressed sentiment dataset to date, with the largest scale and spatiotemporal granularity. However, our dataset also has several limitations. We recommend reading this section before using the dataset.

First, this dataset is constructed based on the geotagged posts only, which accounts for approximately 1–2% of the total traffic at Twitter^21^. This is worth noting when representing the entire Twitter dataset using geotagged tweets that constitute a relatively small proportion. Researchers face the inevitable trade-off between the comprehensive representativeness of tweets and the need for location information (Li *et al*.^49^). For this study, the location information is critical because a major advantage of our sentiment index is the regional variation and the related policy implications. However, a recent paper has found that geotagged posts are subjected to a bias of being happier compared to non-geotagged posts since people like to attach their tweets to a specific location to record the joyous and special events^50^. Nevertheless, our expressed sentiment indices based on the geotagged tweets do have significant correlations with Hedonometer sentiment generated using total tweets (see Table 6), suggesting that geotagged tweets and total tweets have similar sentiment variations over time. Thus, it is recommended that users pay more attention to the local sentiment variations, instead of the absolute sentiment value.

Second, based on the retrieved coordinates of each tweet, we decide to aggregate the tweet sentiment scores at the county/city level to represent the local sentiment instead of going to a finer level. This is due to the precision consideration since the mid of 2019. Twitter changed its policy of location sharing in 2019 to put more emphasis on personal data protection^51^. Instead of sharing the exact coordinates, according to the study, most tweets are sharing the location which can be a country, city, or point of interest. To accommodate this change and prevent the potential bias introduced by policy change, we choose the county/city level as our finest aggregation level.

Third, as we lack socio-demographic information on Twitter users, our database should be regarded as an expressed sentiment database for Twitter users, as opposed to the overall population. We have tested an alternative aggregation approach to mitigate biases towards certain “Tweetaholics”, that involves first aggregating sentiment indices of tweets at the user level, followed by aggregating user sentiment at different administrative regions. We find a high correlation between the indices from different aggregation methods (Table 8). This consistency across aggregation methods bolsters confidence in our TSGI index’s ability to represent Twitter users. To what extent the sentiment trends of Twitter users represent the overall population requires further investigation and validation.

**Table 8:** Correlations of expressed sentiment index with the alternative aggregation approach on different administrative levels.

Admin level	Correlation	p-Value
World	0.996	0.000
Country	0.910	0.000
State/Province	0.948	0.000
County/City	0.957	0.000

## Data Availability

Codes for the raw text processing, model training, and statistics generation are available on our project GitHub (https://github.com/MIT-SUL-Team/Twitter-Sentiment-Geographical-Index).

## References

[1] Diener E, Oishi S, Tay L (2018). Advances in subjective well-being research. Nat Hum Behav.

[2] Jaidka K (2020). Estimating geographic subjective well-being from Twitter: A comparison of dictionary and data-driven language methods. Proc. Natl. Acad. Sci. USA.

[3] Deaton A (2008). Income, health, and well-being around the world: evidence from the Gallup World Poll. J. Econ. Perspect..

[4] Diener E, Chan MY (2011). Happy people live longer: Subjective well-being contributes to health and longevity. Appl. Psychol. Health Well Being.

[5] Selezneva E (2011). Surveying transitional experience and subjective well-being: Income, work, family. Econ. Syst. Res..

[6] Voukelatou V (2021). Measuring objective and subjective well-being: dimensions and data sources. International Journal of Data Science and Analytics.

[7] Lucas RE, Freedman VA, Carr D (2019). Measuring Experiential Well-Being among Older Adults. J. Posit. Psychol..

[8] Schimmack U (2009). Measuring wellbeing in the SOEP. Schmollers Jahrb..

[9] Clark, A. *SWB as a measure of individual well-being*. (Oxford University Press, 2016).

[10] Patrick, S. W. *et al*. Well-being of Parents and Children During the COVID-19 Pandemic: A National Survey. *Pediatrics* **146**, (2020). 10.1542/peds.2020-016824 32709738

[11] Nayak M, Narayan KA (2019). Strengths and weakness of online surveys. IOSR Journal of Humanities and Social Science.

[12] Bail CA (2020). Assessing the Russian Internet Research Agency’s impact on the political attitudes and behaviors of American Twitter users in late 2017. Proc. Natl. Acad. Sci. USA.

[13] Sahoo, S. R. & Gupta, B. B. Real-Time Detection of Fake Account in Twitter Using Machine-Learning Approach. in *Advances in Computational Intelligence and Communication Technology* 149–159 (Springer Singapore, 2021).

[14] Habib MW, Sultani ZN (2021). A Review of Machine Learning Approach for Twitter Sentiment. Analysis. Al-Nahrain Journal of Science.

[15] Passi, K. & Motisariya, J. Twitter Sentiment Analysis of the 2019 Indian Election. in *IOT with Smart Systems* 805–814 (Springer Singapore, 2022).

[16] Schwartz AJ, Dodds PS, O’Neil‐Dunne JPM, Danforth CM, Ricketts TH (2019). Visitors to urban greenspace have higher sentiment and lower negativity on Twitter. People and Nature.

[17] Lyu, X., Chen, Z., Wu, D. & Wang, W. Sentiment Analysis on Chinese Weibo Regarding COVID-19. in *Natural Language Processing and Chinese Computing* 710–721 (Springer International Publishing, 2020).

[18] Chai Y, Kakkar D, Palacios J, Zheng S (2022). Harvard Dataverse.

[19] (2016). Harvard Dataverse.

[20] Wang J (2022). Global evidence of expressed sentiment alterations during the COVID-19 pandemic. Nat Hum Behav.

[21] Qazi U, Imran M, Ofli F (2020). GeoCoV19: a dataset of hundreds of millions of multilingual COVID-19 tweets with location information. SIGSPATIAL Special.

[22] Pradha, S., Halgamuge, M. N. & Tran Quoc Vinh, N. Effective Text Data Preprocessing Technique for Sentiment Analysis in Social Media Data. in *2019 11th International Conference on Knowledge and Systems Engineering (KSE)* 1–8 (ieeexplore.ieee.org, 2019).

[23] Go A, Bhayani R, Huang L (2009). Twitter sentiment classification using distant supervision. CS224N project report, Stanford.

[24] Wisesty, U. N., Rismala, R., Munggana, W. & Purwarianti, A. Comparative Study of Covid-19 Tweets Sentiment Classification Methods. in *2021 9th International Conference on Information and Communication Technology (ICoICT)* 588–593 (2021).

[25] Hinton, G. E. & Salakhutdinov, R. R. Replicated softmax: an undirected topic model. *Adv. Neural Inf. Process. Syst*. **22**, (2009).

[26] Harish, B. S., Guru, D. S. & Manjunath, S. Representation and classification of text documents: A brief review. *IJCA, Special Issue on RTIPPR (2)* 110–119 (2010).

[27] Galke, L. & Scherp, A. Bag-of-Words vs. Graph vs. Sequence in Text Classification: Questioning the Necessity of Text-Graphs and the Surprising Strength of a Wide MLP. in *Proceedings of the 60th Annual Meeting of the Association for Computational Linguistics (Volume 1: Long Papers)* 4038–4051 (Association for Computational Linguistics, 2022).

[28] Araujo, A. *et al*. From Bag-of-Words to Pre-trained Neural Language Models: Improving Automatic Classification of App Reviews for Requirements Engineering. in *Anais do XVII Encontro Nacional de Inteligência Artificial e Computacional* 378–389 (SBC, 2020).

[29] Sun, C., Qiu, X., Xu, Y. & Huang, X. How to Fine-Tune BERT for Text Classification? in *Chinese Computational Linguistics* 194–206 (Springer International Publishing, 2019).

[30] Munikar, M., Shakya, S. & Shrestha, A. Fine-grained sentiment classification using bert. *2019 Artificial Intelligence* (2019).

[31] Pota, M., Ventura, M., Catelli, R. & Esposito, M. An Effective BERT-Based Pipeline for Twitter Sentiment Analysis: A Case Study in Italian. *Sensors* **21**, (2020). 10.3390/s21010133 PMC7796054 33379231

[32] Ndukwe, I. G., Amadi, C. E., Nkomo, L. M. & Daniel, B. K. Automatic Grading System Using Sentence-BERT Network. in *Artificial Intelligence in Education* 224–227 (Springer International Publishing, 2020).

[33] Rudinger, R., May, C. & Van Durme, B. Social Bias in Elicited Natural Language Inferences. in *Proceedings of the First ACL Workshop on Ethics in Natural Language Processing* 74–79 (Association for Computational Linguistics, 2017).

[34] Williams, A., Nangia, N. & Bowman, S. A broad-coverage challenge corpus for sentence understanding through inference. in *Proceedings of the 2018 Conference of the North American Chapter of the Association for Computational Linguistics: Human Language Technologies*, Volume 1 *(Long Papers)*10.18653/v1/n18-1101 (Association for Computational Linguistics, 2018).

[35] Minaee S (2021). Deep Learning–based Text Classification: A Comprehensive Review. ACM Comput. Surv..

[36] Ankit, Saleena N (2018). An Ensemble Classification System for Twitter Sentiment Analysis. Procedia Comput. Sci..

[37] Elfwing S, Uchibe E, Doya K (2018). Sigmoid-weighted linear units for neural network function approximation in reinforcement learning. Neural Netw..

[38] He K, Gkioxari G, Dollar P, Girshick R (2020). Mask R-CNN. IEEE Trans. Pattern Anal. Mach. Intell..

[39] HEAVY.AI. https://www.heavy.ai/.

[40] Mozetič I, Grčar M, Smailović J (2016). Multilingual Twitter Sentiment Classification: The Role of Human Annotators. PLoS One.

[41] Trupthi, M., Pabboju, S. & Narasimha, G. Sentiment Analysis on Twitter Using Streaming API. in *2017 IEEE 7th International Advance Computing Conference (IACC)* 915–919 (ieeexplore.ieee.org, 2017).

[42] Hong L, Convertino G, Chi E (2011). Language Matters In Twitter: A Large Scale Study. ICWSM.

[43] Bae Y, Lee H (2012). Sentiment analysis of twitter audiences: Measuring the positive or negative influence of popular twitterers. J. Am. Soc. Inf. Sci. Technol..

[44] Golder SA, Macy MW (2011). Diurnal and seasonal mood vary with work, sleep, and daylength across diverse cultures. Science.

[45] Elbagir S, Yang J (2019). Twitter sentiment analysis using natural language toolkit and VADER sentiment. Proceedings of the international multiconference of engineers and computer scientists.

[46] Kanakaraj, M. & Guddeti, R. M. R. NLP based sentiment analysis on Twitter data using ensemble classifiers. in *2015 3rd International Conference on Signal Processing, Communication and Networking (ICSCN)* 1–5 (ieeexplore.ieee.org, 2015).

[47] Pennebaker, J. W., Francis, M. E. & Booth, R. J. Linguistic inquiry and word count: LIWC 2001. *Mahway: Lawrence Erlbaum Associates* **71**, 2001 (2001).

[48] Gallagher, R. J., Frank, M. R., Mitchell, L. & Schwartz, A. J. Generalized word shift graphs: a method for visualizing and explaining pairwise comparisons between texts. *EPJ Data* (2021).

[49] Li Z (2021). Measuring global multi-scale place connectivity using geotagged social media data. Sci. Rep..

[50] Jiang, J., Thomason, J., Barbieri, F. & Ferrara, E. Geolocated Social Media Posts are Happier: Understanding the Characteristics of Check-in Posts on Twitter. in *Proceedings of the 15th ACM Web Science Conference 2023* 136–146 (Association for Computing Machinery, 2023).

[51] Zhang, J., DeLucia, A. & Dredze, M. Changes in Tweet Geolocation over Time: A Study with Carmen 2.0. in *Proceedings of the Eighth Workshop on Noisy User-generated Text (W-NUT 2022)* 1–14 (Association for Computational Linguistics, 2022).

